# Exploring the acceptance of robotic assisted surgery among the Indian population: An empirical investigation

**DOI:** 10.12688/f1000research.145052.2

**Published:** 2024-06-27

**Authors:** Smitha Nayak, Vinod C. Nayak, Sathvika G. S.

**Affiliations:** 1 Department of Humanities and Management, Manipal Institute of Technology, Manipal Academy of Higher Education, Manipal, Karnataka, 576104, India; 2 Department of Forensic Medicine and Toxicology, Kasturba Medical College, Manipal Academy of Higher Education, Manipal, Karnataka, 576104, India; 3 Department of Biomedical Engineering, Manipal Institute of Technology, Manipal Academy of Higher Education, Manipal, Karnataka, 576104, India

**Keywords:** robotic-assisted surgery, technology acceptance model, eHealth Literacy, intention, mediation

## Abstract

**Background:**

Technology has completely transformed healthcare, starting with X-ray machines and MRIs to telehealth and robotic surgeries to e-health records. The launch of minimally invasive surgery (MIS) serves as a milestone in medical history, offering benefits such as smaller incisions, shorter hospital stays, and faster recovery, making it a preferred surgical option. This study mainly explores patients’ willingness to adopt robot-assisted surgery (RAS) technology in a surgical intervention and is assessed in the backdrop of the Technology Acceptance Model (TAM).

**Methods:**

This research project employs a post-positivist research philosophy and a cross-sectional research design. A structured, pre-tested questionnaire was used to collect data from 280 respondents.

**Results:**

The results revealed that trust had a significant impact on Perceived Usefulness (β = 0.099) and Perceived Ease of Use (β = .157), and eHealth literacy had a significant impact on Perceived Ease of Use (β = 0.438) and Perceived Usefulness (β = 0.454). Additionally, Perceived Usefulness partially influenced behavioral intention (β = 0.123), and attitude had a significant influence on behavioral intention (β = 0.612). The analysis revealed an insignificant impact of eHealth literacy on Perceived Usefulness (β = 0.067). The Standard Root Mean Square Residual (SRMR) value was <0.8. Mediation analysis also revealed partial mediation between the constructs. The SRMR rating of this model is 0.067, indicating that it fits the data well.

**Conclusion:**

This study revealed that a patient's intention will be high if he or she believes that RAS is beneficial in treating his or her ailment. In comparison, information related to RAS is clearly known, and it does not directly affect selection intention. eHealth literacy is a significant antecedent to patients’ behavioral intention. Hence, the healthcare industry must devise strategies to promote the acceptance of RAS at all levels.

## Introduction

Since its inception, technology has gradually merged with the medical industry. Beginning with inventions like x-ray machines and MRIs and expanding to include telehealth, robotic surgery, and electronic health data, it has radically transformed healthcare. Technology has become an essential component of the medical industry and is responsible for patient diagnosis, treatment, and data administration. The substantial impact of technology on surgical techniques has caused a significant revolution in the field of medicine. Modern technology has improved patient outcomes ( Kim *et al*., 2017), increased precision, decreased invasiveness, and increased the number of therapeutic alternatives ( Prasad, 2013).

Technological advancements in surgery have also been embraced. The launch of minimally invasive surgery (MIS) has served as a milestone in medical history, offering benefits such as shorter hospital stays, minor incisions, and faster recovery, making it a preferred surgical option ( Irani *et al*., 2016). Further technological advancements in MIS have led to the evolution of robotic-assisted surgeons (RAS), which have displayed tremendous advantages to the economy, healthcare system, doctors, and patients ( Bora *et al*., 2020).

The All India Institute of Medical Sciences, New Delhi, received India's first urologic robotic installation in 2006, following the US FDA's approval of the da Vinci system in 2000. Over the next 10 years, robotic surgery in India has seen unprecedented progress. As of July 2019, our country had 66 facilities, 71 robotic installations, and more than 500 skilled robotic surgeons. Over the past 12 years, more than 12,800 surgeries have been performed with robotic assistance ( Bora *et al*., 2020). As per this trend, RAS is expected to expand in India and revolutionize the healthcare system.

Even though there is an increasing trend in the number of RAS globally ( Xue & Liu, 2022) and in India, research reveals that patients’ perceptions of RAS are unclear. Research undertaken by Irani *et al*. (2016) revealed that 34 percent of the participants had a vague understanding of the differences between laparoscopic and open surgery. Furthermore, 46 percent of the participants were not clear regarding the differences between laparoscopic intervention and RAS. Inconsistency in patients’ awareness of the modes of technological interventions was also observed in another study undertaken among female gynecological patients, and Ahn *et al*. (2014). Similar research findings have been documented by Ammer *et al*. (2022) and Chan *et al*. (2022). Differences in understanding and acceptance of RAS were also observed between genders ( McDermott *et al*. 2020).

There has been an increased focus on the co-production of health, because health outcomes are influenced by a complex interplay between physicians, patients, and healthcare systems, rather than being solely determined by medical staff or hospitals ( Puja Turakhia, 2017). There is a need to ensure that patients make informed decisions, which the healthcare system is bound to facilitate. Knowledge of patient preferences and awareness are needed to create an ecosystem that facilitates patients to make informed decisions. This is of paramount importance in the context of RAS and LS, as despite research evidence on the benefits of these interventions, they have gained poor patient acceptance. This could be attributed to the apprehensions that technology has created among patients who traditionally view healthcare as isolated from technology ( Buabbas *et al*., 2020). Against this backdrop, it is important to explore the factors that would explain patients’ intentions to adopt RAS or LS.

The Technology Acceptance Model (TAM) proposed by Davis (1989) serves as the theoretical underpinning for this research endeavor. The “Technology Acceptance Model” (TAM), which sheds light on the crucial roles of perceived usefulness and ease of use in influencing an individual's tendency towards embracing emerging technological breakthroughs, forms an essential component in understanding the dynamics of technology adoption. The importance of TAM lies in its simple explanation of how a person's assessment of a technology's prospective advantages and how user-friendly it is has a significant impact on their preparedness to interact with and employ new technological tools. TAM provides helpful insights into developing and directing the development of user-centric technologies that connect with users and pave the way for their effective adoption and integration into their lives by highlighting the underlying interplay between these aspects. Research has shown ( Xie *et al*., 2022; Xu *et al*., 2021) demonstrates a positive influence of eHealth literacy among patients on health outcomes.

**Figure 1.**
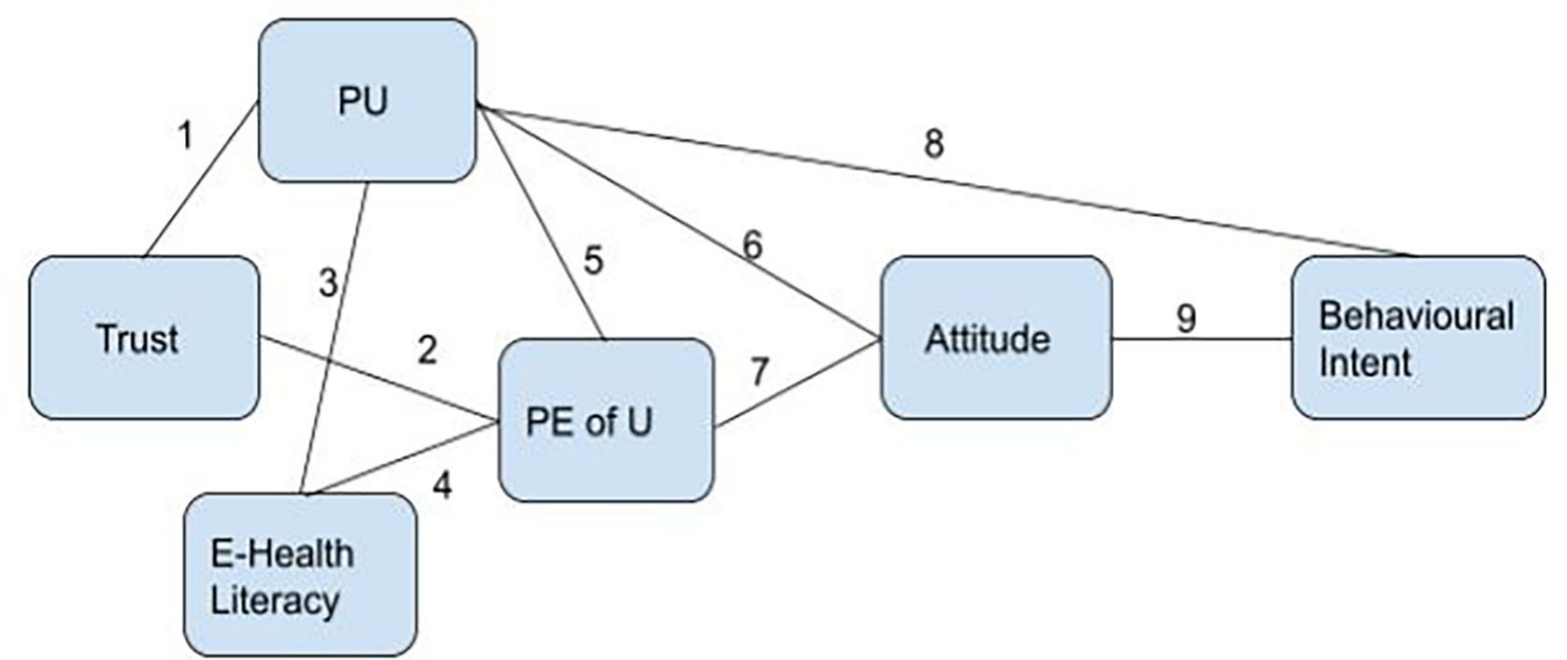
Proposed research framework.

A new era has begun in India, thanks to the quick and broad adoption of the Internet, which has given the populace unparalleled access to a massive library of health-related knowledge ( Parija *et al*., 2020). The increase in digital connectedness has been crucial in enabling people to make wise choices regarding their health and well-being. People throughout the country can now easily access a multitude of medical information, from symptoms and treatments to health tips and preventive measures. In addition to raising awareness, the democratization of health information has made it possible for people to actively participate in their healthcare, have informed conversations with healthcare professionals, and promote their well-being. Thus, the Internet’s quick spread has shown to be an effective catalyst for creating a culture in which people are empowered to take control of their health through informed decision-making. Finding reliable and accurate insights from a sea of knowledge requires a detailed eye. People are more likely to make wise health decisions when they have access to digital literacy ( McCray, 2004). Hence, to ascertain the population’s ability to obtain and utilize health information, this research endeavor has integrated eHealth literacy ( Norman & Skinner, 2006) into the conceptual framework ( Figure 1).

The healthcare environment is significantly shaped by patient trust in doctors, which has implications for patient happiness and medical outcomes. The development of an effective doctor-patient trust connection is important for improving treatment adherence, increasing patient engagement, and eventually improving health outcomes ( Hall *et al*., 2001). Therefore, fostering and preserving patient trust serves as the foundation for efficient and patient-centered medical treatment in an era characterized by increased patient empowerment and information availability ( Krot & Rudawska, 2016). Hence, the patient's trust in the doctor is included as an external construct in the TAM to explore its influence on a person’s intention to opt for RAS. We explored whether patients' trust in the physician influences their beliefs about perceived ease of use and perceived usefulness of RAS.

## Methods

### Study design

This research endeavor was quantitative and used a cross-sectional research methodology. "Post-positivism" is the research philosophy that was taken into account in this study; it deals with the development of empirical approaches to understanding and exploring human behavior. This study considered that surgeons with RAS training would perform the surgery and was founded on a thorough examination of the literature and the theoretical underpinnings of the TAM ( Davis, 1989). Therefore, trust was included as an antecedent in this study. This new era of digital evolution has enhanced e-health literacy, enabling patients to make informed health decisions. Hence, e-health literacy was incorporated into this framework to assess its influence on attitudes towards and intention to adopt RAS.

### Study setting

There is significant research confirmation indicating a compelling association between lower socioeconomic status and health literacy, healthcare use, and health outcomes ( Diez Roux, 2012). This research study was undertaken in Bangalore Urban, Udupi, and Dakshina districts of Karnataka state in India, which are ranked in the top three based on the “Human Development Index Report” published by the Government of Karnataka. Purposive sampling was used to enroll participants from various backgrounds to explore their perceptions of the RAS. Participants were recruited only if they were older than 18 years of age and knew English. Snowball sampling was used to recruit the participants. Respondents were contacted in person and explained by the researchers the purpose of the study and assured that their data would only be used for academic purpose, and if they consented to participate in the survey orally, to the researcher, the questionnaire was given to them in hard copy or in soft copy format. The participants had two options. They could record their response in the hard copy of the questionnaire or using the Google form. As the participants had access to the internet and WhatsApp, all chose to record their responses using the Google form. Hence, the Google form link was shared with the participants, and they were requested to submit their responses. The estimated sample size was 260 ( Hair Jr. *et al*., 2019). Data from 280 participants were collected for the study.

### Data collection

This marketing study was exempt from submission to the Institutional Ethics Committee of Manipal Academy of Higher Education as per clause 6 of the circular titled “Project exemption from submission to IEC” issued by the Institutional Ethical Committee dated 14/1/2021 and hence approval from the department level committee was obtained. Approval was obtained from the Department Review. Committee of the Department of Bio-Medical Engineering, Manipal Institute of Technology, Manipal Academy of Higher Education, India, on June 14 ^th^, 2023. Approval on the research gap, conceptual framework, scales to be adopted in the study and research design to be adopted to by the research team was obtained only after a detailed presentation and proposal submission. After this approval, the research project was implemented. A structured questionnaire was designed to capture the responses. The research instrument was divided into two sections. The first section documented the demographic details of the respondents and the second section captured responses on the research constructs (Perceived Usefulness (PU), Perceived Ease of Use (PEU), Trust, Attitude, Behavioural intention) adapted form ( Kao *et al*., 2022) and eHealth Literacy Scale (eHLS) ( van der Vaart *et al*., 2011). The participants’ responses in the second section were captured on a Likert scale, where 1= strongly disagree and 5 = strongly agree. Data were gathered from July 2023 to August 2023 ( Nayak *et al.,* 2023a, b). IBM SPSS Statistics 27 (RRID: SCR_016479; Armonk, NY: IBM Corp) was used to perform the descriptive analysis, and the results are presented in the subsequent section. SmartPLS 4 (RRID: SCR 022040) was used to evaluate the proposed hypotheses and perform the mediation analysis. Research Project completion approval was obtained from the department on August 15, 2023.

## Results

A total of 280 individuals participated in the study, 126 of whom were men and 154 were women. Only 5% of the respondents had experienced RAS scenarios, while 43% had already undergone surgical operations. Notably, 70% of the participants were aware of RAS ( Table 1).

**Table 1.** Descriptive analysis (N = 280).

Characteristics	Component	N = 280 (%)
**Sex**	Male	126 (45.2)
	Female	154 (54.8)
**Age Group**	18-22	66 (23.3)
	23-42	92 (33)
	43 and above	122 (43.7)
**Field of Education**	Tech & Engineering	112 (39.7)
	Management	89 (31.9)
	Medicine & Health Science	32 (11.5)
	Social Science	29 (10.4)
	Pure Science	18 (6.5)
**Surgical Experience**	Yes	121 (43.01)
	No	159(56.99)
**Robotic-Assisted Surgery Experience**	Yes	14 (5.01)
	No	266 (94.99)
**Aware of Robotic Surgery**	Yes	199 (70.96)
	No	81 (29.04)

**Figure 2.**
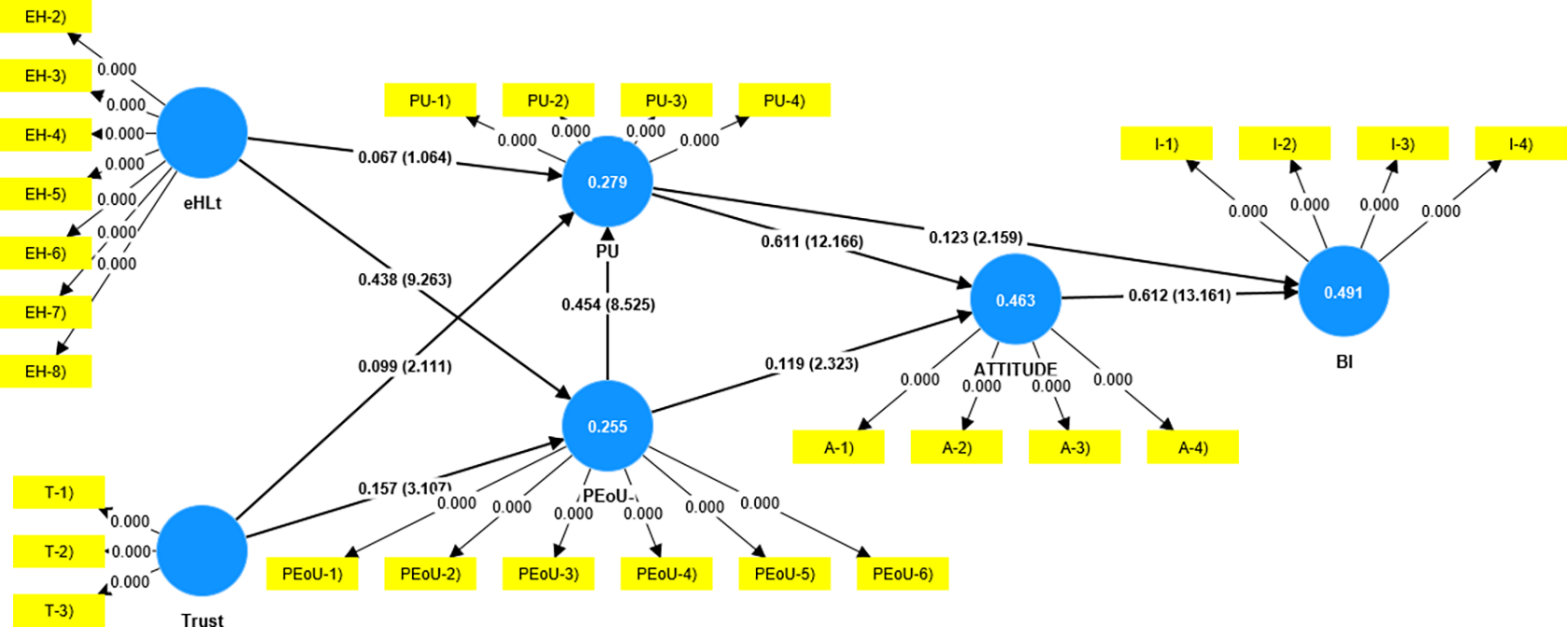
Structural model.

The measurement model was assessed to establish the reliability and validity of the constructs ( Table 2). In the structural model ( Figure 2), the factor loading of the items was examined to ensure that they exceeded 0.5 ( Hair *et al*., 2010). Although ( Vinzi *et al*., 2010) recommended a minimum factor loading of 0.7, it has been noted that researchers frequently run into lower outer loadings in social science studies. It would be better to consider how the removal of indicators might impact the improvement of the reliability and validity of the constructs rather than doing so right away. Additionally, only if doing so increases composite dependability (Average Variance Extracted) above a certain threshold value can one consider deleting indicators with factor loadings ranging from 0.4 and 0.7 ( Sarstedt *et al*., 2017). In the present study, eHLs1 (loading = 0.116) was eliminated because it was far below the threshold value.

**Table 2.** Construct reliability and validity.

Construct	Indicators	Outer loadings	Cronbach’s Alpha	Composite Reliability (rho_a)	Composite reliability (rho_c)	AVE	Outer weights	VIF
Attitude	A1	0.822	0.734	0.773	0.831	0.556	0.402 ***	1.706
	A2	0.845					0.399 ***	1.811
	A3	0.636					0.247 ***	1.326
	A4	0.656					0.268 ***	1.311
eHLs	E2	0.673	0.868	0.876	0.898	0.558	0.143 ***	2.010
	E3	0.696					0.176 ***	2.134
	E4	0.752					0.178 ***	2.035
	E5	0.832					0.190 ***	2.718
	E6	0.794					0.230 ***	2.217
	E7	0.752					0.230 ***	1.728
	E8	0.715					0.187 ***	1.629
	PEoU-1	0.824	0.854	0.854	0.871	0.657	0.252 ***	3.050
	PEoU-2	0.859					0.237 ***	2.986
	PEoU-3	0.603					0.165 ***	3.448
	PEoU-4	0.662					0.174 ***	1.385
	PEoU-5	0.836					0.227 ***	1.510
	PEoU-6	0.769					0.239 ***	2.283
	PU-1	0.844	0.826	0.827	0.884	0.657	0.312 ***	2.025
	PU-2	0.803					0.288 ***	1.828
	PU-3	0.801					0.305 ***	1.688
	PU-4	0.793					0.329 ***	1.594
	T-1	0.833	0.802	0.808	0.883	0.715	0.447 ***	1.511
	T-2	0.868					0.361 ***	2.137
	T-3	0.836					0.376 ***	1.895
	I-1	0.896	0.903	0.912	0.932	0.775	0.283 ***	2.914
	I-2	0.812					0.243 ***	1.980
	I-3	0.908					0.291 ***	3.228
	I-4	0.903					0.316 ***	3.050

*** p<0.01.

** <0.1.

Construct reliability and validity were established using Cronbach's alpha, rho_a, and composite reliability ( Table 2). The computed rho_a value above the cutoff of 0.7 ( Wasko & Faraj, 2005), which is a sign of strong reliability according to ( Henseler *et al*., 2016), fell between the composite reliability as defined by ( Sarstedt *et al*., 2017) and Cronbach's alpha. The “Average Variance Extracted” (AVE) value of more than 0.5 was required to show convergent validity. The Fornell and Larcker methodology was used to confirm discriminant validity in this research ( Table 3).

**Table 3.** Discriminant validity.

Constructs	Attitude	BI	PEoU	PU	Trust	eHLt
Attitude	**0.746**					
BI	0.695	**0.881**				
PEoU	0.433	0.265	**0.765**			
PU	0.672	0.535	0.515	**0.810**		
Trust	0.319	0.385	0.281	0.246	**0.846**	
eHLt	0.375	0.339	0.482	0.314	0.283	**0.747**

Note: The diagonal italic is the square root of AVE. Below the diagonal elements are the correlations between the construct’s values.

The R ^2^ and Q ^2^ path coefficients obtained in the structural model enabled researchers to evaluate the proposed relationships within the research framework. The R ^2^ establishes that the predictive capacity of the model should be above 0.1( Falk and Miller, 1992). Q ^2^ determines the predictive relevance of the model and should be greater than 0. In the current model, both the R ^2^ and Q ^2^ values were above the threshold values, showing the effectiveness of the model ( Table 4). "Standard Root Mean Square Residual (SRMR) was used to measure model fitness. The threshold estimate of SRMR was <0.8 ( Sarstedt *et al*., 2017). This model had a good fit for the data, as indicated by its SRMR value of 0.067. Furthermore, Q ^2^ was calculated if the endogenous construct was more than zero; hence, predictive relevance was established.

**Table 4.** Testing direct relationships.

Hypothesis	Path coefficient	Standard deviation (STDEV)	t value (bootstrap)	P values	F ^2^	BI
H1:Trust -> PU	0.099 **	0.047	2.111	0.035	0.012	(0.003, 0.189)
H2: Trust -> PEoU-	0.157 ***	0.050	3.107	0.002	0.030	(0.057, 0.254)
H3: eHLt -> PU	0.067	0.063	1.064	0.288	0.005	(0.524, 0.706)
H4: eHLt -> PEoU-	0.438 ***	0.047	9.263	0.000	**0.236**	(0.349, 0.532)
H5: PEoU- -> PU	0.454 ***	0.053	8.525	0.000	**0.213**	(0.347, 0.556)
H6: PU -> Attitude	0.611 ***	0.050	12.166	0.000	**0.511**	(0.510, 0.707)
H7: PEoU- -> Attitude	0.119 **	0.051	2.323	0.020	0.019	(0.018, 0.217)
H8: PU -> BI	0.123 **	0.057	2.159	0.031	0.016	(0.009, 0.235)
H9: Attitude -> BI	0.612 ***	0.047	13.161	0.000	**0.403**	(0.524, 0.706)
R ^2^ Attitude = 0.463	Q ^2^ Attitude = 0.155					
R ^2^ BI = 0.491	Q ^2^ BI = 0.137					
R ^2^ PEoU = 0.255	Q ^2^ PEoU = 0.239					
R ^2^ PU = 0.279	Q ^2^ PU = 0.112					

Abbreviation: BI, bias-corrected.

*** p<0.01.

** <0.05.

* <0.1.

The hypothesis-testing results are presented in Table 4. The results revealed that trust had a significant impact on PU (β = 0.099, t = 2.111, p = 0.035) and PEoU (β = .157, t = 3.107, p  = 0.002). eHLt had a significant impact on PEoU (β = 0.438, t = 9.263, p = 0.000), and PEoU had a significant impact on PU (β = 0.454, t = 8.525, p = 0.000). PU (β = 0.611, t = 12.166, p = 0.000) and PEoU (β = 0.199, t = 2.323, p = 0.020) influenced attitude. PU partially influenced BI (β = 0.123, t = 2.159, p = 0.031) and attitude had a significant influence on BI (β = 0.612, t = 13.161, p = 0.000). The analysis revealed an insignificant impact of eHLt on Perceived Usefulness (β = 0.067, t = 1.064, p = 0.288).

### Mediation analysis

In this study, two mediators were proposed and assessed. In the first case, mediation analysis was conducted to assess the mediating role of attitude towards RAS in the relationship between PU and BI ( Table 5). The results revealed a complementary partial mediation of PU (β = 0.413, t = 9.579, p = 0.000) through RAS attitude on behavior intention. The effects of trust and eHLt on PU were assessed using the PEoU. Complementary patrial mediation was observed for trust in PU (β = 0.138, t = 4.162, p = 0.000) through PEoU. Full mediation was observed between eHLt and PU (Î ^2^ = 0.230, t = 6.1919.579, p = 0.000) through PEoU.

**Table 5.** Mediation analysis.

Path	Indirect Effect, p-value	Direct Effect, p-value	Specific Indirect Effect	SD	T Value	p Value	BI (2.5%, 97.5%)	Mediation
PU -> Attitude -> BI	0.536, 0.000	0.123, 0.000	0.413, 0.000	0.043	9.579	0.000	0.334, 0.502	Complementary Partial
Trust -> PEoU -> PU	0.245, 0.000	0.108, 0.017	0.138	0.033	4.162	0.000	0.079, 0.208	Complementary Partial
eHLt -> PEoU -> PU	0.312, 0.000	0.082, 0.198	0.230	0.037	6.191	0.000	0.165, 0.301	Full mediation

### Important-Performance Matrix Analysis (IPMA)

**Figure 3.**
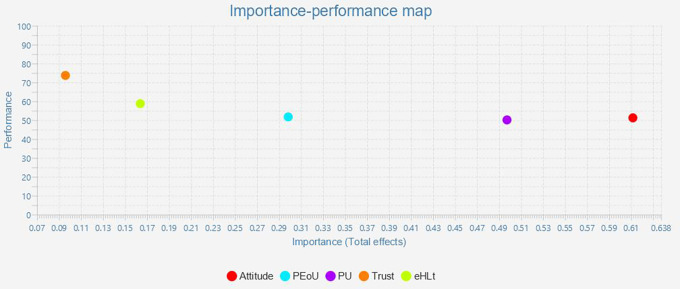
Important-performance matrix analysis.

The outcome of IPMA is presented as bootstrapping with 5,000 subsamples, demonstrating that some of the path coefficients are statistically significant and that all conditions for using the IPMA approach are satisfied. IPMA elucidates the relative significance and performance of exogenous (trust, eHLt, PU, PEoU, and Attitude) and endogenous constructs (behavior intention) about each other. Their significance and efficacy are shown by their aggregate impacts and index values. The impact on the endogenous variables is significant. The intrinsic potential of the latent variable scores is demonstrated through effectiveness. Along the X- and Y-axes, the significance and efficacy were quantified. The entire effect is shown on the x-axis, and the effectiveness is represented on the y-axis. A construct performed better when it had a higher mean value ( Sarstedt *et al*., 2017). The results for the IMPA ( Figure 3 and Table 6). All construct performances ranged from 50 to 70. Furthermore, Attitude (Importance = 0.612 Performance = 51.257), Perceived Usefulness (Importance = 0.498 Performance = 50.165), and Perceived Ease of Use (Importance = 0.299, Performance = 51.748) must be given priority as their importance and performance ( Table 6, Figure 3).

**Table 6.** Important-performance matrix analysis of behavior intention.

Latent construct	Importance (Total effect)	Performance (Index value)
Attitude	0.612	51.257
PEoU	0.299	51.748
PU	0.498	50.165
Trust	0.096	73.724
eHLt	0.164	58.770

## Discussion and Conclusion

The results of this research suggest that patients' understanding of RAS information and subsequent decisions about medical procedures are influenced by the degree of trust they have developed with their doctors **. “**Trust” embedded in their relationship enables patients to comprehend the benefits of PEoU and PU of RAS and enables them to take a rational decision. This is in line with the research evidence presented by Yang & Wu (2018) and, Petrocchi *et al*. (2019) who state that a strong relationship between the healthcare service provider/doctor and patient strengthens health outcomes and enables the patient to make better-informed health decisions. In addition, eHLt was observed to significantly influence PEoU, which is in line with the results of previous studies McCray (2004); Kao *et al*. (2022); Yuan *et al*. (2022). The ease with which a person can use and understand Internet health information can have a significant impact on their perception of medical care. eHealth literate people are better able to obtain accurate and pertinent health information and make wise decisions regarding their well-being. This competency also improves patients' capacity to interact effectively with healthcare professionals, encouraging meaningful dialogue and group decision making. PEoU was observed to positively influence the PU of the RAS. It is evident from this result that if patients can clearly comprehend the benefits of RAS, it will enable them to determine the benefits themselves. The PU construct is borrowed and altered from Kao *et al*. (2022) and Davis (1989). The term originally refers to an information technology system that the user uses personally, and it states that user acceptance will grow for systems that are simple to use. However, in this case, it was a doctor managing RAS. Hence, the construct indicators were altered to suit the study for a better interpretation of the results. In addition, the findings demonstrate that there is no association between eHLt and PU of RAS, even if patients can easily acquire knowledge about RAS and comprehend its function. Although eHLt does not directly affect willingness to use, it still has an impact through PEoU. The impact of eHealth literacy on the PEoU of RAS has significant effects on the Indian population's acceptance and utilization of advanced medical technologies. In this context, eHealth literacy, which encompasses the ability to comprehend and navigate digital health information, is a critical factor in determining individuals' ability to appreciate and accept this mode of surgical intervention. The relationship between eHealth literacy and the perceived ease of use of robot-assisted surgery highlights the importance of tailored educational initiatives to equip people with awareness of RAS. The healthcare industry and its specialists should take advantage of India's extensive digital integration to raise awareness and encourage acceptance among the population. This proactive strategy could substantially impact how people view robot-assisted surgery (RAS), change their attitudes, and increase their willingness to opt for this technology.

It was also observed that PU indirectly influences BI through attitude, which is in line with previously documented literature ( Kao *et al*. 2022; Beldad & Hegner, 2018; Horowitz & Kahn, 2021; Horowitz & Kahn, 2021). It is interesting to note that attitude towards RAS is a significant antecedent to BI; hence, building a positive attitude among the population is necessary for the adoption of RAS.

In conclusion, the use of Robot-Assisted Surgery (RAS) has increased noticeably recently and offers obvious advantages over traditional surgical techniques or endoscopic procedures, especially in complex surgeries at difficult anatomical sites. Since its introduction in India in 2006, a minimum of 12,800 surgeries have been successfully completed using RAS technology. Given the advantages of the RAS mode, the healthcare system should design and implement strategies to enhance awareness and, hence, acceptance among the Indian population. The penetration of the Internet in India has opened up a plethora of means for channelizing communication with the audience ( AlMuammar *et al*., 2021). The healthcare system and healthcare professionals must use different forms of social media to educate the population on the benefits of RAS, enabling them to build a positive attitude toward RAS and, hence, BI. As documented by MINI Tejaswi (2021) RAS is gaining acceptance among patients and healthcare professionals in India, there is a need to further promote positive attitudes among the Indian population to build acceptance of RAS in India.

This study makes two contributions to the literature. Unlike earlier research that primarily concentrated on the efficiency of RAS surgical equipment, this study employed the TAM to evaluate the actions and intentions of RAS among the Indian population. According to this study, an individual's perceptions regarding whether they believe surgery would be useful or whether they have access to enough information to make informed decisions are influenced by their level of trust. In addition, this study aids in the investigation of variables influencing an individual's readiness to undergo sophisticated surgical procedures. This research also investigates the influence of eHLt, which is a significant antecedent to mold positive attitudes, thereby influencing their acceptance of RAS.

Doctors play a crucial and essential role in successfully explaining the benefits of robotic-assisted surgery in patients. Beyond their medical knowledge, they play an important role in converting difficult technical information into clear and understandable language. Doctors may explain how robot-assisted surgery may improve surgical results, reduce invasiveness, speed up recovery, and improve patient experiences by having open and compassionate conversations with patients. This open communication encourages confidence and cooperation between healthcare professionals and patients being treated by them by empowering them to make knowledgeable healthcare decisions. Clinicians act as both advocates and educators, ensuring that patients understand the advantages of this novel surgical approach and may influence their care. This will strengthen the trust a patient displays in the doctor and, hence, influence their behavior.

The literature also identifies important criticisms, such as TAM's shortcomings in identifying issues outside of its purview, such as costs and underlying structural pressures driving users to adopt an invention. The continued adoption and evolution of TAM will depend on how well it is implemented in cutting-edge medical technologies. It is important to note that this early cross-sectional study did not account for differences among other medical specialties, which calls for attention in future research frameworks. Such frameworks could also compare and contrast the preferences and tendencies of patients who had prior experience with robotic surgery (RSS) and those who did not. Future research can provide more thorough insights, producing findings that more accurately reflect heterogeneous patient groups.

## Data Availability

Figshare: Figshare: Exploring the acceptance of robotic assisted surgery among the Indian population: An empirical investigation. https://doi.org/10.6084/m9.figshare.24131124.v1 ( Nayak *et al.*, 2023c) This project contains the following underlying data:
- Data file Figshare: RAS Paper. https://doi.org/10.6084/m9.figshare.25256833.v1 ( Nayak *et al.*, 2024) This project contains the following extended data:
- RAS_Questionnaire Data are available under the terms of the Creative Commons Attribution 4.0 International license (CC-BY 4.0)
