# Supplementary material for: Exploring the acceptance of robotic assisted surgery among the Indian population: An empirical investigation
Source: F1000Res. 2024 Aug 20;13:544. Originally published 2024 May 28. [Version 3] doi: 10.12688/f1000research.145052.3 (PMC11316172; doi:10.12688/f1000research.145052.3)
Supplement: Peer review history

# Reviewer report: Reviewer response for version 2

**DOI:** 10.5256/f1000research.168235.r309499

**Published:** 2024-08-09

**Authors:** Kalayarasan Raja

**Affiliations:** 1 Jawaharlal Institute of Postgraduate Medical Education & Research, , Puducherry,, India

The present review analyzed patients’ willingness to adopt robot-assisted surgery for surgical intervention using the Technology Acceptance Model. Results were based on the data from 280 respondents. Authors found that perceived Usefulness is an important factor in choosing RAS.

 My comments

 1. In the Introduction authors have quoted the data of robotic platforms and surgeries till 2019. If available, they can use the updated data for India.

 2. Discussion and limitations of the study included in the discussion were appropriate.

 3. Authors have proposed the focus of future studies.

Is the work clearly and accurately presented and does it cite the current literature?

Yes

If applicable, is the statistical analysis and its interpretation appropriate?

Yes

Are all the source data underlying the results available to ensure full reproducibility?

Yes

Is the study design appropriate and is the work technically sound?

Yes

Are the conclusions drawn adequately supported by the results?

Yes

Are sufficient details of methods and analysis provided to allow replication by others?

Yes

Reviewer Expertise:

Robotic and laparosocpic gastrointestinal surgery

I confirm that I have read this submission and believe that I have an appropriate level of expertise to confirm that it is of an acceptable scientific standard.

# Response

**Published:** 2024-08-12

**Authors:** smitha nayak

**Affiliations:** Manipal University, India

Dear Reviewer, 

 Thank you for the comments. We have incorporated the changes in the manuscript, wherever possible and the updated version is uploaded for your approval. 

 The introduction section and the direction for future research have been updated as suggested by you.

 Thank you,

 Research team

# Reviewer report: Reviewer response for version 2

**DOI:** 10.5256/f1000research.168235.r296301

**Published:** 2024-06-28

**Authors:** Nripesh Kumar Nrip

**Affiliations:** 1 Department of Computer Application, Bharati Vidyapeeth Institute of Management, Kolhapur, Maharashtra, India; 2 Department of Computer Applications, Bharati Vidyapeeth (Deemed to be University), Pune, Maharashtra, India

No major corrections ware suggested in the first review and the authors have amended their article with minute suggested changes. 

 Approved from Reviewer's view.

Is the work clearly and accurately presented and does it cite the current literature?

Yes

If applicable, is the statistical analysis and its interpretation appropriate?

Yes

Are all the source data underlying the results available to ensure full reproducibility?

Yes

Is the study design appropriate and is the work technically sound?

Yes

Are the conclusions drawn adequately supported by the results?

Yes

Are sufficient details of methods and analysis provided to allow replication by others?

Yes

Reviewer Expertise:

Multidisciplinary, Specially Information Technology in various domain of Human and Society like Agriculture, Health care, Education etc.

I confirm that I have read this submission and believe that I have an appropriate level of expertise to confirm that it is of an acceptable scientific standard.

# Reviewer report: Reviewer response for version 1

**DOI:** 10.5256/f1000research.158938.r285902

**Published:** 2024-06-11

**Authors:** Nripesh Kumar Nrip

**Affiliations:** 1 Department of Computer Application, Bharati Vidyapeeth Institute of Management, Kolhapur, Maharashtra, India; 2 Department of Computer Applications, Bharati Vidyapeeth (Deemed to be University), Pune, Maharashtra, India

**Title:** **Exploring the acceptance of robotic-assisted surgery among the Indian population: An empirical investigation**

**Authors:** Smitha Nayak, Vinod C. Nayak, Sathvika G. S.

**Abstract and Introduction:** The abstract provides a concise summary of the study, highlighting the key findings and significance of the research. The introduction effectively sets the stage by discussing the integration of technology in healthcare and the specific advancements brought by robotic-assisted surgery (RAS). It frames the research within the context of the Technology Acceptance Model (TAM) and emphasizes the importance of understanding patient perceptions and eHealth literacy in the adoption of RAS.

**Comments:**

- The background information is comprehensive and well-researched.
- The rationale for the study is clearly articulated.
- The objectives are well-defined and relevant 

**Methods:** The methodology section is detailed, describing the cross-sectional research design and the use of a structured questionnaire to gather data from 280 respondents. The incorporation of TAM and eHealth literacy into the conceptual framework is well-explained.

**Comments:**

- The sample size is appropriate for the study.
- The use of both hard copy and digital questionnaires is commendable for flexibility.
- The inclusion criteria and recruitment process are clearly described.
- The statistical tools and techniques used (IBM SPSS and SmartPLS) are appropriate for the analysis.

**Results:** The results are presented with clarity, supported by tables and figures. The study successfully establishes the reliability and validity of the constructs. The findings on the impact of trust, perceived ease of use (PEoU), perceived usefulness (PU), and eHealth literacy on behavioral intention are well-documented.

**Comments:**

- The use of structural modeling to evaluate the hypotheses is appropriate and well-executed.
- The results are statistically significant and contribute to the understanding of factors influencing the acceptance of RAS.
- The mediation analysis adds depth to the findings.

**Discussion:** The discussion section effectively interprets the results, linking them back to the theoretical framework and previous research. The emphasis on the role of trust and eHealth literacy in shaping patient attitudes and behavioral intentions is insightful.

**Comments:**

- The discussion is thorough and provides practical implications for the healthcare industry.
- The recommendations for promoting RAS acceptance through enhanced communication and education are well-founded.
- The potential influence of digital literacy on the adoption of advanced medical technologies is well-articulated.

**Conclusion:** The conclusion succinctly summarizes the key findings and underscores the importance of trust and eHealth literacy in the acceptance of RAS. The study’s contributions to the literature and practical applications are clearly stated.

**Comments:**

- The conclusion is concise and effectively wraps up the study.
- The emphasis on the role of healthcare professionals in educating patients about RAS is a valuable insight.

**Overall Assessment:** This study makes significant contributions to the understanding of factors influencing the acceptance of robotic-assisted surgery among the Indian population. The integration of TAM and eHealth literacy provides a robust framework for analyzing patient behavior. The findings are well-supported by rigorous data analysis and offer practical recommendations for healthcare practitioners and policymakers.

**Minor Suggestions:**

- Ensure consistency in the use of abbreviations (e.g., PU, PEoU, RAS) throughout the manuscript.

**Final Decision:** Accept with minor revisions.

Is the work clearly and accurately presented and does it cite the current literature?

Yes

If applicable, is the statistical analysis and its interpretation appropriate?

Yes

Are all the source data underlying the results available to ensure full reproducibility?

Yes

Is the study design appropriate and is the work technically sound?

Yes

Are the conclusions drawn adequately supported by the results?

Yes

Are sufficient details of methods and analysis provided to allow replication by others?

Yes

Reviewer Expertise:

Multidisciplinary, Specially Information Technology in various domain of Human and Society like Agriculture, Health care, Education etc.

I confirm that I have read this submission and believe that I have an appropriate level of expertise to confirm that it is of an acceptable scientific standard.

# Response

**Published:** 2024-06-21

**Authors:** smitha nayak

**Affiliations:** Manipal University, India

Thank you for reviewing the article. Your suggestions have indeed added value to the manuscript. I will respond to the review comments and as per your suggestions, I will resubmit the article after incorporating the suggested changes. I will ensure uniformity is maintained. 

 Regards, 

 Prof. Smitha Nayak
